# Orbital T-cell lymphoma in youngest recorded patient – early diagnosis, management, and successful outcome: a case report and review of the literature

**DOI:** 10.1186/s13256-018-1630-2

**Published:** 2018-05-14

**Authors:** Hind Manaa Alkatan, Saleh Hamad Alrashed, Ammar C. Al-Rikabi, Yasser H. Al-Faky

**Affiliations:** 1 Department of Ophthalmology, College of Medicine, King Saud University-Medical City, Riyadh, Saudi Arabia; 2 Department of Pathology, College of Medicine, King Saud University-Medical City, Riyadh, Saudi Arabia; 3 College of Medicine, King Saud University, Riyadh, Saudi Arabia

**Keywords:** T-cell lymphoma, Orbit, Chemotherapy, Lymphoproliferative, Case report

## Abstract

**Background:**

Primary orbital peripheral T-cell lymphoma, not otherwise specified is an exceedingly rare disorder with a very poor outcome, and to the best of our knowledge only a few cases have been reported in the English literature. We present the youngest reported case describing the successful outcome after management with a thorough review of the English literature of all the reported cases of primary peripheral T-cell lymphoma, not otherwise specified.

**Case presentation:**

Our patient is a 3-year-old Syrian boy who presented with gradual progressive orbital swelling. A physical examination showed a left orbital dystopia and a superior medial displacement of the globe. Extraocular motility was limited in upward elevation of his left eye. A computed tomography scan and magnetic resonance imaging of his orbit showed a mass involving the lateral and inferior walls of his left orbit and extending intraconally. A diagnosis of peripheral T-cell lymphoma, not otherwise specified was made by careful histopathological examination and Berlin-Frankfurt-Munster protocol was initiated. A 6-month follow up with orbital magnetic resonance imaging showed no sign of orbital or brain involvement.

**Conclusions:**

Through this report we emphasize two takeaway lessons: (1) always have a high level of suspicion of this entity regardless of the age of the patient; and (2) careful histopathological examination is very important for prompt confirmation of the diagnosis and early commencement of proper treatment.

**Electronic supplementary material:**

The online version of this article (https://doi.org/10.1186/s13256-018-1630-2) contains supplementary material, which is available to authorized users.

## Highlights

In this case report we describe the youngest patient to have a rare type of lymphoma occurring in the orbit recorded in the English literature. We discuss the challenging histopathological diagnosis, the importance of ophthalmologists’ awareness to include this entity in their differential diagnosis, and the promising resolution with proper treatment.

## Background

Peripheral T-cell lymphoma, not otherwise specified (PTCL-NOS) is a subtype of the aggressive group of peripheral T-cell lymphomas (PTCLs), which are derived from various types of mature T-cells, that do not meet the criteria for the other specifically defined subtypes of PTCL [1]. Primary orbital PTCL-NOS is an extremely rare disorder. To the best of our knowledge only a few cases have been reported in the English literature and out of these the youngest case was reported in 2012 by Amit *et al*. involving a 6-year-old boy [2–6]. We present the youngest reported case with histopathological confirmation and show initial successful outcome with a 1-year follow up since early diagnosis and proper management. The case has been prepared according to the guidelines for medical case reports described by Gagnier *et al*. (CARE = case report) [7].

## Case presentation

The parents of a 3-year-old Syrian boy noticed that he had gradual progressive painless swelling of his left lower eyelid and ptosis over the course of 2 months that significantly increased over the last 2 weeks. There was no significant loss of vision. Medically, the parents recalled loss of appetite of 1-week duration, with no history of trauma, other systemic manifestations, or past surgery. He had an unremarkable family history.

**Fig. 1:**
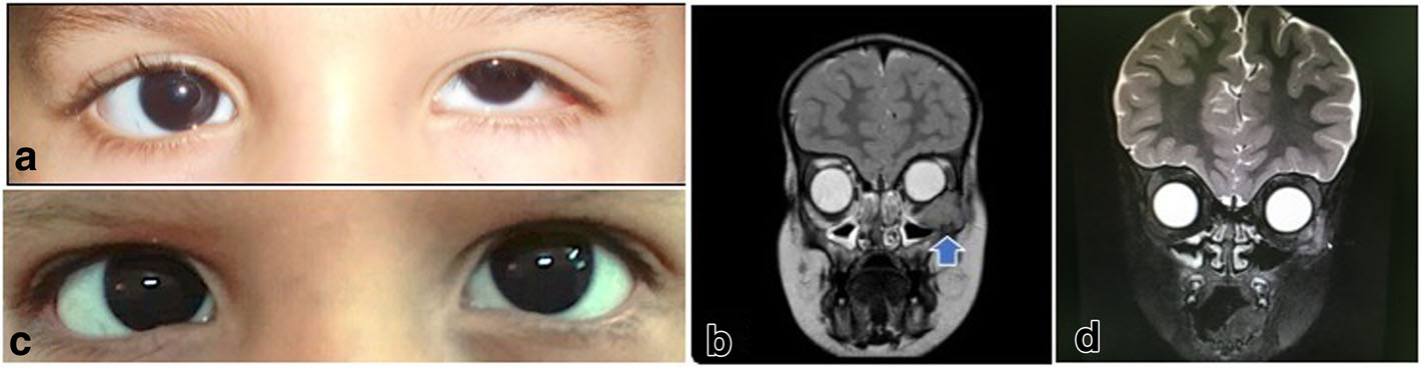
**a** The clinical appearance of the left displaced globe with narrowing of the palpebral fissure and pseudoptosis. **b** The large orbital mass lesion along the inferior and lateral orbital bony wall (*arrow head*) on T2-weighted magnetic resonance image at initial presentation. **c** The appearance of the left eye with resolution of the swelling and the dystopia 9 months after the initiation of chemotherapy. **d** Repeated T2-weighted magnetic resonance image more than 6 months after treatment with resolved left orbital mass

An ophthalmological examination revealed left orbital dystopia and superomedial displacement of the globe. Extraocular motility was limited in upward elevation of his left eye. His left upper lid seemed to be drooping; however, it proved to be pseudoptosis, which was caused by the upward displacement of the globe due to the mass effect. Correspondingly, the palpebral fissure measurement in his left eye was 5 mm, while it measured 9 mm in his right eye (Fig. 1a). Further examination of his left eye revealed a hard lesion involving the inferior orbital rim and the lateral aspect of the orbital cavity, measuring 3.5 cm horizontally by 1.5 cm vertically. There was no proptosis, overlying skin changes, or palpable lymphadenopathy.

A computed tomography (CT) scan revealed a 9.5 mm osseous destruction at the left inferior lateral orbital margin that was associated with a large soft tissue mass measuring 1.9 × 2.1 × 1.9 cm. The mass extended into his left orbital cavity. A small soft tissue component measuring 7.8 × 7.8 × 9.7 mm was also seen extending toward the subcutaneous zygomatic region. Magnetic resonance imaging (MRI) of his orbits showed a solid enhancing mass lesion, involving the lateral and inferior walls of his left orbit, and extending into the intraconal compartment. The mass was displacing the globe superiorly and medially with medial displacement of the left inferior rectus muscle (Fig. 1b). His optic nerve appeared normal and there was no associated mass effect on his right globe. No brain abnormalities were demonstrated.

**Fig. 2:**
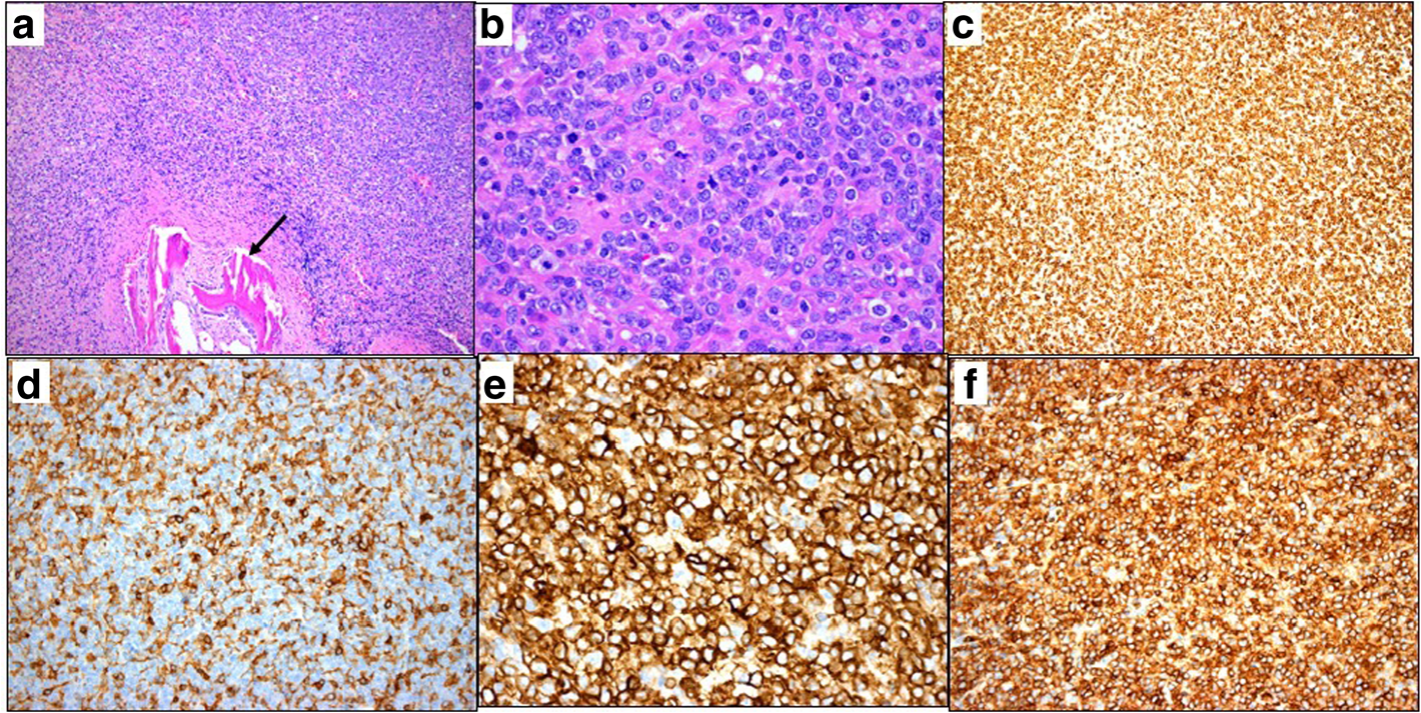
**a** The histopathologic appearance of the lesion showing diffuse sheets of atypical lymphocytic cells infiltrating the adjacent bone (*black arrow*). (Original magnification × 100, hematoxylin and eosin.) **b** Higher power of the lymphocytic infiltrate with frequent mitotic figures. (Original magnification × 400, hematoxylin and eosin.) **c** Prominent staining of the diffuse sheets of T lymphocytes with the T-cell marker. (Original magnification × 100, CD3.) **d** The cells also showed less strong expression for CD4. (Original magnification × 200.) **e** Staining of the T lymphocytes with CD8. (Original magnification × 200.) **f** Similarly, the cells showed strong positive staining with CD43. (Original magnification × 200)

An incisional biopsy under general anesthesia was performed and a 2.1 × 1.5 × 1.0 cm tissue sample was obtained. Histological slides showed diffuse effacement atypical cells of variable size infiltrating the bone (Fig. 2a). The proliferating cells often had clear cytoplasm, resembling Reed–Sternberg cells, with broad cytological spectrum ranging from irregular, pleomorphic, hyperchromatic to vesicular nuclei and prominent nucleoli. Many mitotic figures were seen (Fig. 2b). The histological features were consistent with non-Hodgkin’s lymphoma. The immunohistochemical (IHC) stains showed that the tumor cells were strongly positive for T-cell markers including CD3, CD4, CD8, and CD43 (Fig. 2c–f). Stains for B cell markers were all negative: CD20, CD21, CD30, CD56m, and granzyme B as a marker for natural killer (NK) cells. Stain for terminal deoxynucleotidyl transferase (TdT) as a marker for non-Hodgkin’s lymphoblastic lymphoma was also negative. Formalin-fixed, paraffin-embedded (FFPE) 10 μm tumor sections were used to extract DNA using the QIAamp DNA Mini Kit(DNA FFPE Tissue Kit; by "OR from" QIAGEN). Qualitative polymerase chain reaction (PCR) was done for T-cell receptors (TCR beta and TCR gamma) gene rearrangement. The test showed positivity for clonal TCR gene rearrangement. Polyacrylamide gel electrophoresis results also confirmed the presence of a heavy chain TCRB positive band consistent with T-cell rearrangement. These results confirmed the diagnosis of PTCL-NOS.

Serology for Epstein–Barr virus (EBV), cytomegalovirus, and human immunodeficiency virus were negative. A systemic evaluation including: CT of his chest, abdomen, and pelvis; bone marrow biopsy; and physical examination of the ear, nose, and throat, were normal. A cardiac function assessment as well as a complete body gallium scan were performed and were negative for metastasis. His peripheral blood workup was within normal limits. He was a high risk and was started on Berlin-Frankfurt-Munster protocol, with 2 weeks on and 2 weeks off chemotherapy. The protocol includes the following agents: cytarabine (ARA-C), L-asparaginase (ASP), cyclophosphamide (CTX), dexamethasone (DEX), doxorubicin (DXR), mercaptopurine (6-MP), methotrexate (MTX), prednisone (PDN), 6-thioguanine (6-TP), and vincristine (VCR). However, the doses and details of administration are beyond the scope of this case report since we are highlighting the challenge and the importance of accurately diagnosing such rare cases. He was followed up every month for 6 months. He tolerated the chemotherapy well according to his parents and his oncologist. He showed remarkable response to chemotherapy with resolution of his signs and symptoms (Fig. 1c). MRI of his orbit was repeated 6-month post-treatment and showed no signs of recurrence of the tumor (Fig. 1d). The outcome was considered to be successful following his 18-month treatment plan and this was documented clinically and by repeated MRI. He was last seen in the clinic in early January 2018 (approximately 20 months after his initial presentation) and was doing well (Additional file 1).

## Discussion and conclusions

Orbital and ocular adnexal lymphoid neoplasms are not scarce, representing 6 to 8% of all orbital tumors, whereas out of the extranodal non-Hodgkin’s lymphomas, primary non-Hodgkin’s lymphoma of the orbit represented 8 to 10% [8, 9]. Based on case series, the most reported orbital lymphoid tumor was B cell non-Hodgkin malignant lymphoma [8, 10]. In 1998, Coupland analyzed 112 cases of ocular adnexal lymphoproliferative disorder, T-cell lymphoma was encountered in only three cases [10]. These cases were all systemic [10]. PTCL in general is an aggressive neoplasm with a poor outcome. Primary orbital PTCL-NOS of the orbit has been rarely reported with, to the best of our knowledge, only six cases being reported in the English literature including ours [2–6] (Table 1).

**Table 1:** Summary of the five previously reported primary orbital cases of peripheral T-cell lymphoma, not otherwise specified and our case

Case #	Author	Age (y)	Sex	Initial presentation	IHC markers	Treatment	Outcome
1)	Coupland *et al*. (1999) [2]	76	M	Subconjunctival masses, right eye. Right anterior orbital mass (by radiology)	CD45, CD3, CD4, CD8	Conjunctival/eyelid – surgical excision Orbital – local irradiation of 36 Gy	**Alive** 5 years after treatment
2)	Lee *et al*. (2006) [3]	61	F	Left eyeball pain. Headache	T-cell markers, UCHL-1	Refused chemotherapy and radiotherapy	**Died** in a month
3)	Janatpour *et al*. (2007) [4]	44	M	Right eye diplopia and retro-orbital pain	CD2, surface CD3, CD45RO, CD43, CD4, CD8, TIA-1 granzyme B	CHOP – eight cycles	**Alive** with resolved masses 3 years after initial visit and treatment
4)	Chen *et al*. (2009) [5]	34	F	Right orbital painful mass. Protruding right eye	CD3 and CD2	Radiotherapy	3-month follow up – proptosis and the protruding mass in the right eye failed to achieve satisfactory resolution
5)	Amit *et al*. (2012) [6]	6	M	Left eye proptosis. Lagophthalmos	CD45, CD45RO, CD3	Radiotherapy Chemotherapy – vincristine and adriamycin	**Died** within 1.5 months of diagnosis
6)	Present case	3	M	Left lower eyelid swelling and ptosis	CD3, CD4, CD8, and CD43	Chemotherapy	**Alive** for 12 months after treatment

*CHOP* Cyclophosphamide, Hydroxyldaunorubicin, Vincristine (Oncovin), Prednisone, *M* male, *F* female, *IHC* immunohistochemical

According to the World Health Organization (WHO) classification of lymphoid neoplasms, the diagnosis of PTCL-NOS requires the integration of morphology, aberrant T-cell phenotype, and clonally rearranged TCR genes [1]; having said that, the diagnosis of PTCL-NOS can still be challenging and can be confused with other PTCLs. In 2011 Weisenburger *et al*. published an extensive review of 340 cases to better understand PTCL-NOS [11]. The 340 cases of PTCL-NOS were diagnosed by a local pathologist from each institution by means of immunophenotypic, cytogenetic, and molecular genetic studies following the criteria of WHO classification [11]. To observe the accuracy of the diagnosis, they drew a panel of four expert hematopathologists from the local site and regional centers to reach a first diagnosis, which was purely based on histopathology, the immunophenotype, and molecular genetic data and a second diagnosis based on the above in addition to complete clinical data. The agreement of the consensus diagnosis of PTCL-NOS by the first group without clinical data was 71% as per their review, while the agreement rate with the consensus diagnosis of PTCL-NOS after additional complete clinical data reached 75%. Disagreements with the consensus diagnosis included other diagnoses: angioimmunoblastic T-cell lymphoma and anaplastic large cell lymphoma (ALCL), anaplastic lymphoma kinase (ALK)-negative. On the other hand, the change of diagnosis from another entity to PTCL-NOS occurred in 14 cases, the commonest out of these were cases of angioimmunoblastic T-cell lymphoma, where clinical information about other manifestations such as skin rash and hypergammaglobulinemia had an influence on the diagnosis [11]. The median age in that large study was 60 years, the majority (around 70%) presented with an advanced disease, thus showing nodal and/or extranodal manifestations such as: hepatosplenomegaly, skin/subcutaneous tissue lesions, and lung involvement. The male-to-female ratio was 1.9:1 [11]. If we analyze our reviewed cases, including ours, the age ranged between 3 years and 76 years with a median of 39 years since two of the cases occurred in childhood and the male-to-female ratio was similarly 2:1. In 2008, Savage published a report differentiating PTCL-NOS from ALK-negative ALCL clinically and immunophenotypically [12]. The cells in PTCL-NOS more frequently expressed CD2, CD3, CD4, and CD43, while they less often expressed epithelial membrane antigen (EMA) or cytotoxic proteins than ALK-negative ALCL, while ALK-negative ALCL tumors were more likely to be positive for cytotoxic markers [12]. In European Society for Medical Oncology Clinical Practice Guidelines, having the following IHC criteria was more in favor with the diagnosis of PTCL-NOS: positive cells to CD4, CD8 markers; a drop-off of CD5 and CD7; and TCR rearrangement of αβ and γδ [13, 14].

In the review of the five reports, not all the cases seem to have a detailed immunohistochemistry. Having said that, based on the previous five authors’ reports, the IHC staining in most of the cases proved T-cell type of lymphoma where tumor cells expressed T-cell markers CD3, CD4, and CD8. One case showed a drop-off of CD5 and CD7. On the other hand, in almost all cases reported, the cells failed to express CD30 and CD56 markers that aid in the diagnosis of ALCL and NK cell lymphoma, respectively. Only four cases had PCR showing TCR rearrangement.

Our case of a 3-year-old child diagnosed with primary orbital PTCL is unusual in the sense that he is the youngest case of primary PTCL-NOS of the orbit ever reported to the best of our knowledge. The presentation of our patient with pseudoptosis was also unusual. The consistency of a hard mass clinically and the appearance of the lesion by imaging gave an impression of a bony origin, such as Ewing sarcoma. However, histopathology revealed PTCL-NOS features. The IHC stains showed the expression of the tumor cells for CD30, CD56, and ALK were negative excluding the diagnosis of ALCL and NK cell lymphoma. In addition, the findings of TCR rearrangements on PCR did confirm our diagnosis of PTCL-NOS despite being challenging and confusing. The overall 5-year survival has been reported to be as low as 32%. Significant numbers of EBV-positive B cells, CD56 expression, and CD30 expression by more than 20% of the tumor cells are bad prognostic factors [11]. Fortunately, the lymphocytic cells in our case failed to express CD56 and CD30. Therefore, even though the follow up in our case is relatively short (1 year), we are hoping for a better survival especially with our early therapeutic intervention.

In conclusion, we have presented the youngest case of proven PTCL-NOS ever reported in the English literature. Our case was unusual in presentation and imaging features; thus, the diagnosis was totally unexpected. The early accurate diagnosis allowed for proper promising treatment. Therefore, we strongly recommend the following: (1) a high level of suspicion of such entities regardless of the age or presentation; and (2) careful histopathological examination and proper tissue diagnosis by an experienced pathologist, which is critical in confirming the diagnosis for early intervention.

## Additional file

Additional file 1: Timeline of the patient's clinical history starting from the date of presentation to the date of last follow up. (JPEG 172 kb)
