# Supplementary figures and images for: Orbital T-cell lymphoma in youngest recorded patient – early diagnosis, management, and successful outcome: a case report and review of the literature
Source: J Med Case Rep. 2018 May 14;12:139. doi: 10.1186/s13256-018-1630-2 (PMC5950188; doi:10.1186/s13256-018-1630-2)

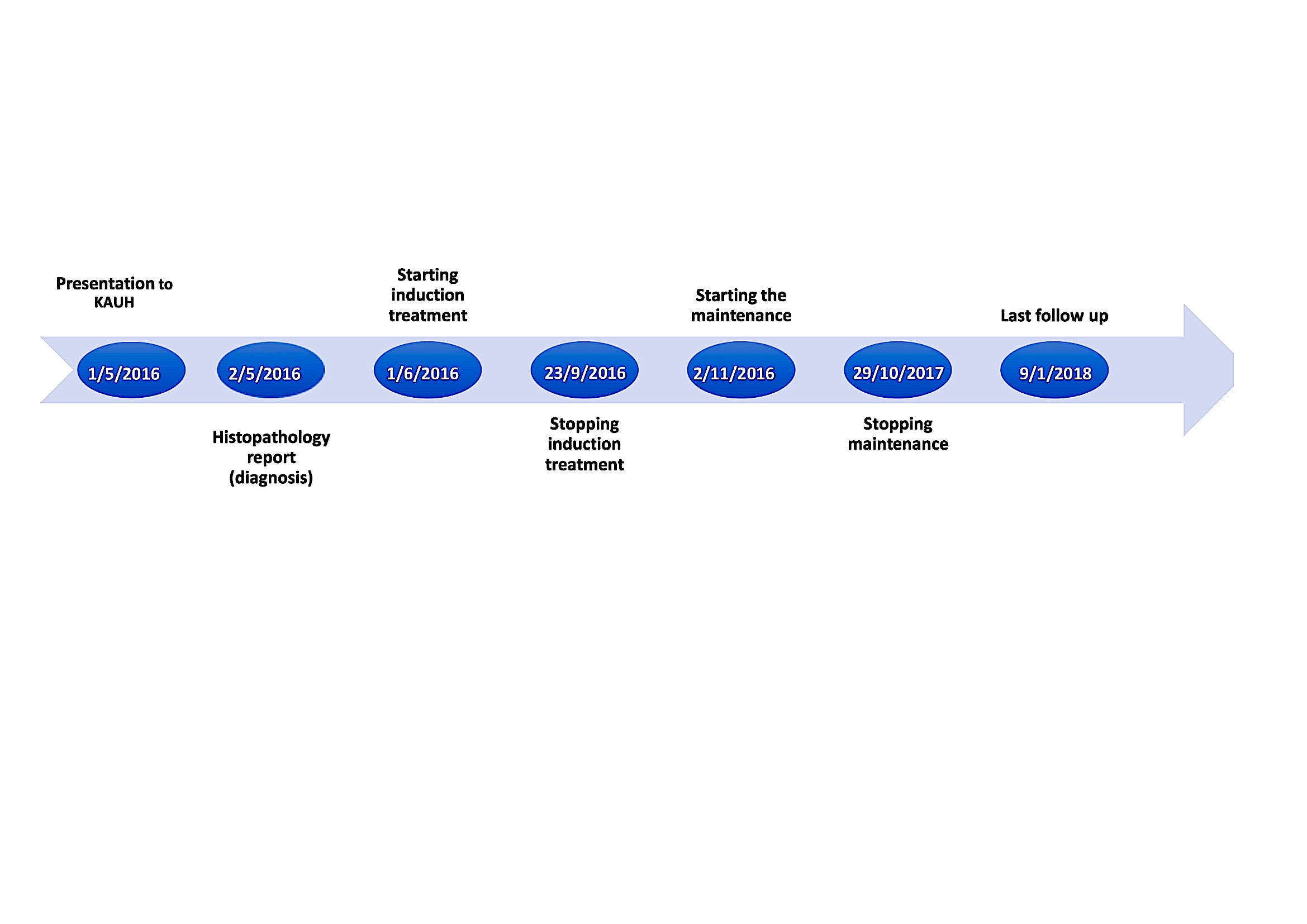

Supplement: Supplementary file 1 — Timeline of the patient's clinical history starting from the date of presentation to the date of last follow up. (JPEG 172 kb) [file 13256_2018_1630_MOESM1_ESM.jpg]
